# Serum adiponectin as a biomarker for in vivo PPARgamma activation and PPARgamma agonist-induced efficacy on insulin sensitization/lipid lowering in rats

**DOI:** 10.1186/1471-2210-4-23

**Published:** 2004-10-18

**Authors:** Baichun Yang, Kathleen K Brown, Lihong Chen, Kevin M Carrick, Lisa G Clifton, Judi A McNulty, Deborah A Winegar, Jay C Strum, Stephen A Stimpson, Gregory L Pahel

**Affiliations:** 1 Departments of Molecular Pharmacology, GlaxoSmithKline, Research Triangle Park, NC 27709, USA; 2 Metabolic Diseases, GlaxoSmithKline, Research Triangle Park, NC 27709, USA; 3 Quantitative Expression, GlaxoSmithKline, Research Triangle Park, NC 27709, USA

## Abstract

**Background:**

PPARγ agonists ameliorate insulin resistance and dyslipidemia in type 2 diabetic patients. Adiponectin possesses insulin sensitizing properties, and predicts insulin sensitivity of both glucose and lipid metabolism. In diet-induced insulin resistant rats and ZDF rats, the current studies determined the correlation between PPARγ agonist-upregulated fatty acid binding protein(FABP3) mRNA in adipose tissue and PPARγ agonist-elevated serum adiponectin, and the correlation between PPARγ agonist-elevated serum adiponectin and PPARγ agonist-mediated efficacy in insulin sensitization and lipid lowering.

**Results:**

Parallel groups of SD rats were fed a high fat/sucrose (HF) diet for 4 weeks. These rats were orally treated for the later 2 weeks with vehicle, either PPARγ agonist GI262570 (0.2–100 mg/kg, Q.D.), or GW347845 (3 mg/kg, B.I.D). Rats on HF diet showed significant increases in postprandial serum triglycerides, free fatty acids (FFA), insulin, and area under curve (AUC) of serum insulin during an oral glucose tolerance test, but showed no change in serum glucose, adiponectin, and glucose AUC. Treatment with GI262570 dose-dependently upregulated adipose FABP3 mRNA, and increased serum adiponectin. There was a positive correlation between adipose FABP3 mRNA and serum adiponectin (r = 0.7350, p < 0.01). GI262570 dose-dependently decreased the diet-induced elevations in triglycerides, FFA, insulin, and insulin AUC. Treatment with GW347845 had similar effects on serum adiponectin and the diet-induced elevations. There were negative correlations for adiponectin versus triglycerides, FFA, insulin, and insulin AUC (For GI262570, r = -0.7486, -0.4581, -0.4379, and -0.3258 respectively, all p < 0.05. For GW347845, r = -0.6370, -0.6877, -0.5512, and -0.3812 respectively, all p < 0.05). In ZDF rats treated with PPARγ agonists pioglitazone (3–30 mg/kg, B.I.D.) or GW347845 (3 mg/kg, B.I.D.), there were also negative correlations for serum adiponectin versus glucose, triglycerides, FFA (for pioglitazone, r = -0.7005, -0.8603, and -0.9288 respectively; for GW347845, r = -0.9721, -0.8483, and -0.9453 respectively, all p < 0.01).

**Conclusions:**

This study demonstrated that (a) PPARγ agonists improved insulin sensitivity and ameliorated dyslipidemia in HF fed rats and ZDF rats, which were correlated with serum adiponectin; (b) Serum adiponectin was positively correlated with adipose FABP3 mRNA in GI262570-treated rats. These data suggest that serum adiponectin can serve as a biomarker for both *in vivo *PPARγ activation and PPARγ agonist-induced efficacy on insulin resistance and dyslipidemia in rats.

## Background

Type 2 diabetes mellitus (T2D) and the metabolic syndrome are characterized by resistance to the action of insulin in peripheral tissues, including skeletal muscle, liver, and adipose. Activation of the peroxisome proliferator-activated receptor gamma (PPARγ) improves insulin sensitivity and lowers circulating levels of glucose, triglycerides and free fatty acids without stimulating insulin secretion in rodent models of T2D [1,2]. PPARγ agonists also alleviate peripheral insulin resistance in humans, and have been effectively used in treatment of T2D patients [3-5]. Fatty acid binding protein(FABP3), adipocyte lipid binding protein(aP2) and lipoprotein lipase (LPL)are response genes of PPARγ and are indicators for in vivo PPARγ activation in adipose tissue [6-9].

Adiponectin, an adipose-specific plasma protein, possesses insulin sensitizing and anti-atherogenic properties [10]. It has been well documented that plasma adiponectin is lower in obese subjects than in lean subjects, lower in diabetic patients than in non-diabetic patients [10-13], and is negatively correlated with body weight, visceral fat mass, and resting insulin level [11,12]. Hotta et al also reported that adiponectin decreased in parallel with the progression of T2D in rhesus monkeys, and there is a strong correlation between plasma adiponectin and systemic insulin sensitivity [14]. Studies by Maeda et al showed that adiponectin knockout mice developed hyperglycemia and hyperinsulinemia while on HF diet, which was reversed by adenoviral-mediated adiponectin expression [15]. Exogenous adiponectin also lowered hepatic glucose production during a pancreatic euglycemic clamp [16], and increased post-absorptive insulin-mediated suppression of hepatic glucose output [10]. The PPARγ agonist, class of insulin sensitizer, has the marked effect of up-regulating serum adiponectin. Combs et al reported that the PPARγ agonist rosiglitazone increased plasma adiponectin in *db/db *mice [17]. Yang et al reported rosiglitazone increased plasma levels of adiponectin in type 2 diabetic patients [18]. Tschritter et al analyzed the associations between plasma adiponectin and insulin sensitivity and serum lipid parameters in nondiabetic individuals, and concluded that plasma adiponectin predicts insulin sensitivity of both glucose and lipid metabolism [19].

While PPARγ agonists increase plasma adiponectin and adiponectin levels predict insulin sensitivity, there is not a clear demonstration of the relationships among PPARγ agonist-increased adiponectin and PPARγ agonist-mediated efficacy on insulin sensitivity/in vivo PPARγ activation. Therefore, the current studies were designed to define these relationships and assess serum adiponectin as a biomarker for in vivo PPARγ activation and PPARγ agonist-induced efficacy on insulin sensitization and lipid lowering.

## Results

### High fat/sucrose (HF) diet induced changes in SD rats

Rats on the HF diet for 4 weeks showed marked insulin resistance and dyslipidemia, indicated by significant increases in postprandial serum levels of triglycerides, free fatty acids, insulin, and area under curve (AUC) for serum insulin during OGTT. But the HF diet did not cause changes in postprandial serum glucose or OGTT glucose AUC compared with rats on normal diet, consistent with an insulin resistant, pre-diabetic phenotype. Serum adiponectin level in rats on HF diet was slightly higher than that in normal diet rats at week 2, but back to the same level at week 4 (Table 1).

**Table 1:** HF diet induced changes in SD rats.

	**Normal diet**	**HF diet**
Triglyceride (mg/dL)		
Prior to start	95.0 ± 8.7	115.6 ± 13.7
2 weeks diet	100.4 ± 7.6	446.2 ± 38.7**++
4 weeks diet	121.7 ± 9.1	388.1 ± 44.5**++
Free fatty acid (mEq/L)		
Prior to start	0.36 ± 0.05	0.41 ± 0.04
2 weeks diet	0.38 ± 0.05	0.56 ± 0.04**++
4 weeks diet	0.24 ± 0.02	0.67 ± 0.06**++
Glucose (mg/dL)		
Prior to start	166.8 ± 5.3	162.8 ± 4.3
2 weeks diet	170.0 ± 11.3	174.1 ± 2.5
4 weeks diet	176.6 ± 2.8	167.0 ± 4.0
Post-prandial insulin (ng/ml)		
Prior to start	0.71 ± 0.11	1.06 ± 0.15
2 weeks diet	1.26 ± 0.29	2.72 ± 0.47**++
4 weeks diet	1.23 ± 0.22	2.39 ± 0.35**++
Insulin AUC during OGTT		
4 weeks diet	241.6 ± 19.5	528.6 ± 84.9**
Glucose AUC during OGTT		
4 weeks diet	7360 ± 416	7533 ± 496
Serum adiponectin (μg/ml)		
Prior to start	3.59 ± 0.25	3.53 ± 0.25
2 weeks diet	3.64 ± 0.32	4.96 ± 0.41*
4 weeks diet	3.75 ± 0.40	4.35 ± 0.40

*p < 0.05 vs Before diet, **p < 0.01 vs Before diet, ++p < 0.01 vs Normal diet.

### PPARγ agonist on adiponectin in SD rats

**Figure 1:**
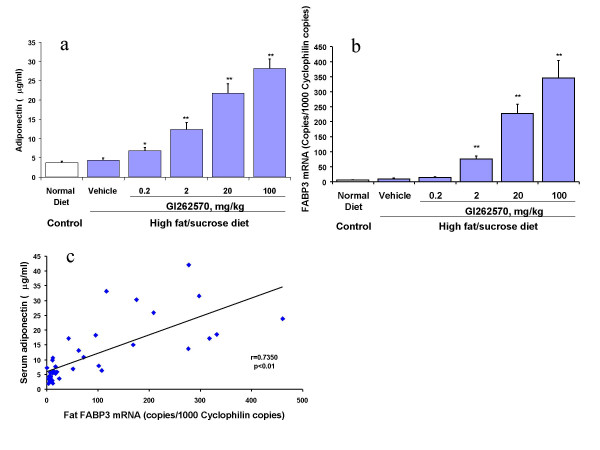
Efeects of PPARγ agonist GI262570 on serum adiponectin level (a), adipose FABP3 mRNA level (b), and the correlation between serum adiponectin and adipose FABP3 mRNA. SD rats were on HF diet for 4 weeks. GI262570 was oral dosed for the later 2 weeks. Mean ± SEM. N = 5–8 in each group. *p < 0.05 vs vehicle. **p < 0.01 vs vehicle.

As showed in Fig. 1, treatment of SD rats on HF diet with GI262570 for 2 weeks dose-dependently increased serum adiponectin, and upregulated adipose FABP3 mRNA without effect on housekeeper genes 18S, β-actin, and cyclophilin. There was a positive correlation between adipose FABP3 mRNA and serum adiponectin (Pearson Correlation Coefficients 0.7350, p < 0.01). A marked increase in serum adiponectin was also observed in GW347845-treated HF fed SD rats (30.93 ± 0.45 vs 4.86 ± 0.30 μg/ml in vehicle. p < 0.01).

### PPARγ agonist-increased serum adiponectin and PPARγ agonist-mediated efficacy on insulin sensitivity and lipid lowering

**Figure 2:**
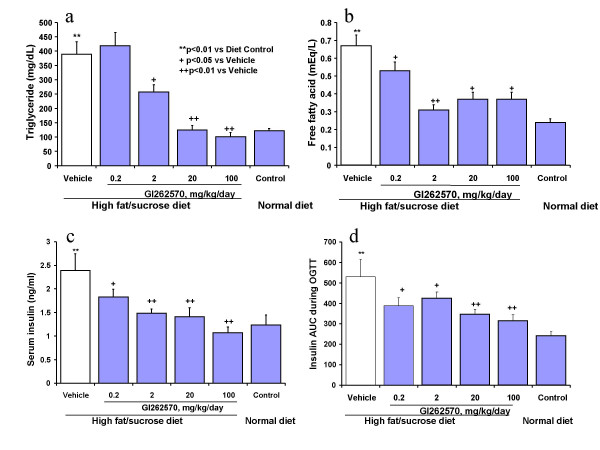
Effects of PPARγ agonist GI262570 on serum insulin, triglycerides, free fatty acids, and insulin AUC during OGTT. SD rats were on HF diet for 4 weeks. GI262570 was oral dosed for the later 2 weeks. Mean ± SEM. N = 7–9 in each group.

**Figure 3:**
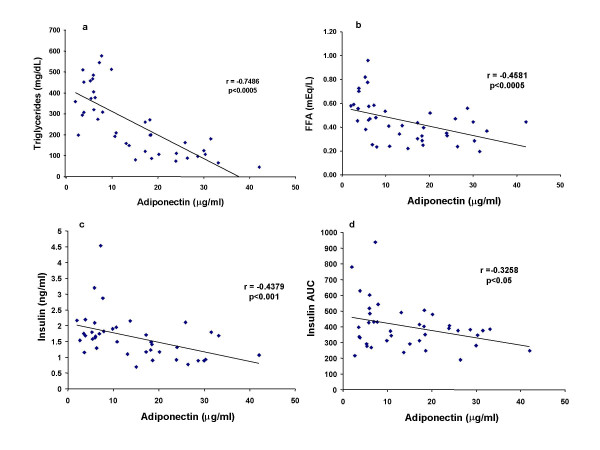
Correlation between PPARγ agonist GI262570 (0.2–100 mg/kg)-elevated serum adiponectin and GI262570-decreased serum insulin, triglycerides, free fatty acids, and insulin AUC during OGTT in HF fed SD rats.

Treatment of rats on HF diet with GI262570 for 2 weeks significantly decreased the diet-induced elevations in postprandial serum triglycerides, free fatty acids, insulin, and insulin AUC in a dose-dependent manner (Fig. 2). Treatment with GW347845 showed a qualitatively similar effect to that of GI262570 treatment (Table 2). There were negative correlations for adiponectin versus triglycerides, free fatty acids, insulin, and insulin AUC (For GI262570, r = -0.7486, -0.4581, -0.4379, and -0.3258; p < 0.005, 0.005, 0.01 and 0.05 respectively, Fig. 3; For GW347845, r = -0.6370, -0.6877, -0.5512, and -0.3812, p < 0.01, 0.01, 0.01 and 0.05 respectively, Table 2).

**Table 2:** Effect of GW347845 (3 mg/kg, B.I.D.) in rats on HF diet.

	Triglycerides (mg/dL)	FFA (mEq/L)	Serum Insulin (ng/ml)	Insulin AUC (min × ng/ml)
Normal diet	98.6 ± 8.6	0.26 ± 0.03	1.34 ± 0.19	241.0 ± 22.8
Diet-Vehicle	455.6 ± 94.2**	0.65 ± 0.07**	1.88 ± 0.16*	356.9 ± 25.3**
Diet-GW347845	139.4 ± 14.8++	0.37 ± 0.03++	1.16 ± 0.08++	267.9 ± 34.2
Corr. Coeff.	-0.637	-0.6877	-0.5512	-0.3812
Vs adiponectin	p < 0.01	p < 0.01	p < 0.01	p < 0.05

Corr. Coeff.: Pearson Correlation Coefficient. *p < 0.05 vs Normal diet. **p < 0.01 vs normal diet. ^++^p < 0.01 vs diet-vehicle. N = 7–8 in each group.

### PPARγ agonists in Zucker rats

Compared with Zucker lean rats, ZDF rats had higher serum insulin, glucose, TG, FFA, but similar serum adiponectin levels. Treatment of ZDF rats with PPARγ agonist pioglitazone or GW347845 for 2 weeks resulted in significantly lower serum glucose, triglycerides, free fatty acids, and modestly lower serum insulin, compared to vehicle treatment. Both pioglitazone and GW347845 markedly increased serum adiponectin in ZDF rats (Table 3). There were also negative correlations for serum adiponectin versus glucose, TG, FFA (for pioglitazone, r = -0.7005, -0.8603, and -0.9288 respectively; for GW347845, r = -0.9721, -0.8483, and -0.9453 respectively, all p < 0.01).

**Table 3:** Effect of pioglitazone and GW347845 in ZDF rats.

	Insulin (ng/ml)	Glucose (mg/dL)	Triglycerides (mg/dL)	FFA (mEq/L)	Adiponectin (μg/ml)
ZDF lean rats					
Vehicle	0.3 ± 0.1	158 ± 4	82 ± 7	0.31 ± 0.02	10.2 ± 0.5
ZDF rats					
Vehicle	2.9 ± 0.5**	525 ± 25**	912 ± 97**	0.62 ± 0.03**	10.0 ± 1.1
Pioglitazone (mg/kg, B.I.D)					
3	2.5 ± 0.6^+^	214 ± 64^+^	251 ± 81^++^	0.26 ± 0.09^++^	44.0 ± 7.7^++^
10	2.8 ± 0.5	154 ± 17^++^	129 ± 19^++^	0.16 ± 0.01^++^	60.0 ± 1.5^++^
30	2.3 ± 0.4^+^	154 ± 9^++^	129 ± 16^++^	0.14 ± 0.01^++^	63.0 ± 0.8^++^
GW347845 (mg/kg, B.I.D)					
3	1.7 ± 0.2^++^	147 ± 6^++^	95 ± 10^++^	0.12 ± 0.01^++^	66.1 ± 0.6^++^

**p < 0.01 vs ZDF lean rats. ^+ ^p < 0.05 vs Vehicle-treated ZDF rats. ^++^p < 0.01 vs Vehicle-treated ZDF rats. N = 6–12 in each group.

## Discussion

Adiponectin possesses insulin sensitizing and anti-atherogenic properties [10]. In most clinical reports, primate studies, and genetic models, serum adiponectin level had been reported to be negatively correlated with body weight, visceral fat mass, and resting insulin level [10-13]. The present study showed that rats fed a HF diet had significantly higher serum insulin and lipids with in 2 weeks, which indicates insulin resistance. However, serum adiponectin level was not decreased by the diet up to 4 weeks. We have subsequently kept rats on the HF diet for up to 20 weeks, and observed a slight increase (instead of decrease) in serum adiponectin level (data not shown). Our data may suggest that the HF diet-induced insulin resistance happened much early than diet-induced change in serum adiponectin. Our data is consistent with studies by Naderali EK et al [19]. In their report, 16 weeks of high fat/glucose diet resulted in significantly higher body weight, fat pad masses, plasma leptin, and higher plasma level of adiponectin, besides higher levels of plasma TG and FFA.

PPARγ is a member of the PPAR family of the nuclear receptor superfamily [6]. PPARγ agonists increase insulin sensitivity and circulating adiponectin [1,2,17,18]. The response genes of PPARγ for in vivo PPARγ activation include LPL, AP2 and FABP3 [6,7,9]. The current study demonstrated that as in other species the PPARγ agonist GI262570 upregulated serum adiponectin level and adipose FABP3 mRNA level in SD rats in a dose-dependent manner. Interestingly, there is a positive correlation between PPARγ full agonist-upregulated serum adiponectin level and adipose FABP3 mRNA level, demonstrating the serum adiponectin level could be a biomarker for in vivo PPARγ activation.

We did perform parallel experiments to check mRNA levels of PPARγ response genes FABP3, aP2 and LPL in epididymal fat. We found that basal level of FABP3 mRNA was very low compared to aP2 and LPL (FABP3:LPL:aP2 = ~1:250:2500), and that PPARγ agonist GI262570 dose-dependently increased FABP3 mRNA. AP2 was abundant in epididymal fat tissues, and was only slightly increased by GI262570 in a non-dose-dependent manner (data not shown). LPL was decreased in high fat diet fed rats, which was reversed by GI262570 but not dose-dependently (data not shown). With in vivo chronic exposure, the effect of PPARγ agonists on gene expression is difficult to separate from the effects on differentiation. In general we find aP2 a better marker of adipocyte differentiation than PPARγ activation. Since PPARγ agonist-mediated action in vivo may vary with organs/tissues (such as liver vs fat; subcutaneous fat vs omental or epididymal fat) [20,21] and duration of treatment, all PPARγ response genes may not be changed in the same manner in one tissue following chronic treatment. Therefore the authors consider that the dose-dependently GI262570 upregulated FABP3 mRNA in epididymal fat caught in the present study is of value for quantitative in vivo PPARγ activation. Thus the correlation data using FABP3 mRNA is of value.

Adiponectin has been demonstrated to have an insulin sensitizing effect [10]. Circulating adiponectin levels were positively correlated with insulin sensitivity, measured both by an euglycemic-hyperinsulinemic clamp and estimated by an oral glucose tolerant test, were negatively correlated with fasting lipids [22]. The PPARγ agonist rosiglitazone increased plasma level of adiponectin, decreased fasting plasma glucose and HBA_1C_, and ameliorated insulin resistance in type 2 diabetic patients [18]. However, the relationship between PPARγ agonist-increased circulating adiponectin and PPARγ agonist-induced efficacy on insulin resistance has not been studied. The current study showed that PPARγ agonists increased serum levels of adiponectin, ameliorated insulin resistance and lipid profile in both diet-induced insulin resistant rats and ZDF rats. There is a correlation between PPARγ agonist-increased serum adiponectin level and PPARγ agonist-induced efficacy in insulin sensitivity/lipid lowering. These data provide a link between PPARγ agonist-elevated circulating adiponectin level and PPARγ agonist-mediated efficacy in insulin sensitivity and lipid lowering, and indicate that serum adiponectin level could be a biomarker for in vivo PPARγ efficacy.

Other adipokines, such as leptin, are important in obesity and insulin resistance. Unlike adiponectin, leptin is positively correlated with fat amount, mass and percentage [23]. It has been reported that PPARγ agonists inhibit the expression and function of leptin [24,25]. Our unpublished study showed that high fat diet resulted in insulin resistance and higher serum leptin level in rats. Treatment of these insulin resistant rats with PPARγ agonist GW7845 improved insulin sensitivity, but did not affect serum leptin level. Therefore leptin is not considered to be a marker for PPARγ efficacy. There are indices for in vivo PPARγ activation (i.g., adipose FABP3 mRNA), or for in vivo PPARγ efficacy on insulin sensitization (i.g., serum insulin and glucose). These indices can not be used to represent both in vivo PPARγ activation and in vivo PPARγ efficacy on insulin sensitization. It is well known that circulating adiponectin increases insulin sensitivity [10], is decreased in T2D patients [10-13], and is negatively correlated with insulin resistance [22]; PPARγ agonists increase insulin sensitivity as well as circulating adiponectin [17,18]. The correlations, serum adiponectin vs adipose FABP3 mRNA and serum adiponectin vs insulin/lipids, in our study demonstrated that serum adiponectin is a good biomarker for both in vivo PPARγ activation and in vivo PPARγ efficacy on insulin sensitization.

## Conclusions

These studies demonstrated that in both diet-induced and genetic rat models of insulin resistant (metabolic) syndrome the full PPARγ agonists GI262570, GW347845, and pioglitazone significantly elevated serum adiponectin levels, increased adipose transcription of the PPARγ response gene FABP3, and were efficacious as expected. This is the first demonstration of correlation among PPARγ agonist-increased serum adiponectin, PPARγ agonist response gene mRNA, and PPARγ agonist-mediated efficacy in insulin sensitivity and lipid lowering. These data indicate that serum adiponectin can serve as a biomarker for both in vivo PPARγ activation and PPARγ agonist-induced efficacy in rats.

## Methods

### Experimental animal and protocols

All procedures performed were in compliance with the Animal Welfare Act and U.S. Department of Agriculture regulations, and were approved by the GlaxoSmithKline Animal Care and Use Committee. Male caesarian derived Sprague Dawley rats (SD, 225–250 g) (Charles River, Indianapolis, IN) were fed rodent chow Purina 5001 (Harlan Teklad, Indianapolis, IN). Male Zucker diabetic fatty (ZDF) and male Zucker lean rats (8 weeks old) (Genetic Models, Indianapolis, IN) were fed Formulab Diet 5008 (PMI Feeds, Richmond, IN). After an adaptation period of 1 week, SD rats were fed a HF diet (TD88137, Containing 34.146% sucrose. 42% of calories from fat. Harlan Teklad, Indianapolis, IN) for 4 weeks. SD Rats fed chow Purina 5001 served as normal diet control. SD rats on HF diet were treated with vehicle (0.5% hydroxypropyl methylcellulose and 0.1% Tween 80), PPARγ agonist GI262570 [7,26-28] (0.2, 2, 20, or 100 mg/kg, QD), or PPARγ agonist GW347845 (3 mg/kg, BID) for the last 2 weeks. ZDF rats were gavaged twice daily for 14 days with vehicle, PPARγ agonist pioglitazone [4] (3, 10, or 30 mg/kg), or PPARγ agonist GW347845 (3 mg/kg) [29,30]. Zucker lean rats were gavaged twice daily for 14 days with vehicle. One day prior to the end of dosing (after 13 days of dosing), serum was obtained from tail vein of SD rats for determining postprandial levels of glucose, insulin, triglycerides, free fatty acids, and adiponectin. The SD rats were then implanted with a jugular cannula. Oral glucose tolerant tests (OGTT) were performed in these SD rats after 14 days of dosing. At the end of the study, SD rats were euthanized with CO_2_. White adipose tissue (WAT, epididymal fat pad) were saved for determining mRNA levels of PPARγ response gene FABP3. In Zucker rats, serum was collected after 2 weeks of dosing for determining postprandial levels of glucose, insulin, triglycerides, free fatty acids, and adiponectin. Zucker rats were then euthanized with CO_2_.

### Determination of postprandial serum chemicals

Serum glucose, triglycerides, and free fatty acids were measured using Ilab600 Clinical Chemistry System (Instrumentation Laboratory).

### Determination of serum adiponectin

Serum adiponectin of SD rats was determined by using adiponectin RIA kit (Linco Research, MO), according to the manufacture's instruction. Serum adiponectin of ZDF rats was determined by using adiponectin ELISA kit (B-Bridge International, CA), according to the manufacture's instruction.

### Jugular vein cannulation

Under anesthesia with isoflurane, surgical site was prepared using standard aseptic technique (with Hiboclens^® ^Chlorhexidine Gluconate, Zeneca Pharmaceuticals, Delaware). A longitudinal incision was made over the right external jugular vein. 5–10 mm of the vein was exposed by blunt dissection. Jugular cannula (Access™ Technologies, IL) was inserted into the vein for about 1 inch. The cannula was secured using sterile sutures. The cannula was routed subcutaneously, exteriorized between the scapulae. The cannula was then filled with dextrose-heparin solution (50:50), and heat sealed.

### OGTT

Rats implanted with jugular cannula were fasted overnight. The following morning, dextrose (0.5 g/ml in water, 2 g/kg body weight) was administered by oral gavage. Blood samples (0.3 ml/time) were obtained from the jugular cannula before gavage, 10, 20, 30, 45, 60, 90 and 120 min after gavage. Blood glucose was immediately measured by using Elite^® ^XL Glucometer (Bayer, Tarrytown, NY). Serum was collected for insulin measurement. Area under curves (AUCs) for glucose and insulin during OGTT were calculated by using WinNonlin™ Noncompartmental Model 200.

### Determination of insulin level

Serum insulin of SD rats level was determined using Rat Insulin ELISA kit (Crystal Chem Inc, IL), according to the manufacture's instruction. Serum insulin level of ZDF rats was determined using Igen's M-SERIES M-8 Analyzer (Igen International, Inc., Gaithersburg, MD).

### Determination of FABP3 mRNA level in white adipose tissue by real time PCR

Total RNA in epididymal fat pad was isolated by the TRIZOL^® ^method [31]. All RNA samples were DNased using the DNA-*free*™ kit (Ambion – according to protocol). The samples were then quantitated by RiboGreen™ (Molecular Probes – according to protocol). GAPDH gene expression was analyzed in the absence of reverse transcriptase to ensure the samples were free of genomic DNA. The samples were then converted to cDNA using the High Capacity cDNA Archive Kit (Applied Biosystems – according to protocol). Samples were diluted to a final concentration of 5 ng/ul of cDNA. PCR results were generated using the 5' nuclease assay (TaqMan) [32] and the ABI 7900 Sequence Detection System (Applied Biosystems, Foster City, CA). Primers and probe for FABP3 are: Forward-GTCGTGACACTGGACGGAGG; Reverse-TTCCCATCACTTAGTTCCCGTG; Probe-CAGAAGTGGGACGGGCAGGAGACTACG. The primers and probe for Cyclophilin are: Forward-TATCTGCACTGCCAAGACTGA; Reverse-CCACAATGCTCATGCCTTCTTTCA; Probe-CCAAAGACCACATGCTTGCCATCCA. A master mixture was utilized which included 900 nM each of the forward and reverse primers, 100 nM probe, and 1 × PCR master mix (Applied Biosystems). The PCR reaction consisted of 12.5 ng of cDNA in a 12.5 ul total reaction volume. The PCR cycling conditions were 95°C for 10 minutes, and 40 cycles of 95°C for 15 seconds and 60°C for 1 minute.

### Statistical analysis

There was a minimum of 5 rats for each data point. Data are presented as mean ± SEM. Correlation between two parameters and the significant level of correlation were analyzed by Pearson correlation analysis. Differences between vehicle and treated groups were analyzed by two-way ANOVA. P less than 0.05 was taken to be significant.

## List of abbreviations

QD: Once a day

BID: Twice a day

PCR: Polymerase Chain Reaction

GAPDH: Glyceraldehyde-3-Phosphate Dehydrogenase

ANOVA: Analysis of Variance

## Authors' contributions

BY is the principal investigator. LC, LGC, JM, and DW participated in the in vivo experiments. KC and JS performed the real time PCR. KB, SS and GP participated in study design and manuscript preparation.

## References

[B1] Brown KK, Henke BH, Blanchard SG, Cobb JE, Mook R, Kaldor I, Kliewer SA, Lehmann JM, Lenhard JM, Harrington WW, Novak PJ, Faison W, Binz JG, Hashim MA, Oliver WO, Brown HR, Parks DJ, Plunket KD, Tong W, Menius JA, Adkison K, Noble SA, Willson TM (1999). A novel N-aryl tyrosine activator of peroxisome proliferator-activated receptor-gamma reverses the diabetic phenotype of the Zucker diabetic fatty rat. Diabetes.

[B2] Willson TM, Brown PJ, Sternbach DD, Henke BR (2000). The PPARs: From orphan receptors to drug discovery. J Medicinal Chem.

[B3] Virtanen KA, Hallsten K, Parkkola R, Janatuinen T, Lonnqvist F, Viljanen T, Ronnemaa T, Knuuti J, Huupponen R, Lonnroth P, Nuutila P (2003). Differential effects of rosiglitazone and metformin on adipose tissue distribution and glucose uptake in type 2 diabetes subjects. Diabetes.

[B4] Hirose H, Kawai T, Yamamoto Y, Taniyama M, Tomita M, Matsubara K, Okazaki Y, Ishii T, Oguma Y, Takei I, Saruta T (2002). Effects of pioglitazone on metabolic parameters, body fat distribution, and serum adiponectin levels in Japanese male patients with type 2 diabetes. Metabolism.

[B5] Pavo I, Jermendy G, Varkonyi TT, Kerenyi Z, Gyimesi A, Shoustov S, Shestakova M, Herz M, Johns D, Schluchter BJ, Festa A, Tan MH (2003). Effect of pioglitazone compared with metformin on glycemic control and indicators of insulin sensitivity in recently diagnosed patients with type 2 diabetes. J Clin Endocrino Metabolism.

[B6] Desvergne B, Wahli W (1999). Peroxisome proliferator-activated receptors: Nuclear control of metabolism. Endocrine Rev.

[B7] Yang B, Clifton LG, McNulty JA, Chen L, Brown KK, Baer PG (2003). Effects of a PPARgamma agonist, GI262570, on renal filtration fraction and nitric oxide level in conscious rats. J Cardiovasc Pharmacol.

[B8] Pearson SL, Cawthorne MA, Clapham JC, Dunmore SJ, Holmes SD, Moore GBT, Smith SA, Tadayyon M (1996). The thiozolidinedione insulin sensitiser, BRL 49653, increases the expression of PPARgamma and aP2 in adipose tissue of high-fat-fed rats. Biochemical & Biophysical Research Communications.

[B9] Way JM, Harrington WW, Brown KK, Gottschalk WK, Sundseth SS, Mansfield TA, Ramachandran RK, Willson TM, Kliewer SA (2001). Comprehensive messenger ribonucleic acid profiling reveals that peroxisome proliferator-activated receptor gamma activation has coordinate effects on gene expression in multiple insulin-sensitive tissues. Endocrinology.

[B10] Berg AH, Combs TP, Scherer PE (2002). ACRP30/adiponectin: an adipokine regulating glucose and lipid metabolism. TRENDS Endocrinol Metabolism.

[B11] Hotta K, Matsuzawa Y (2001). [Molecular mechanism in the development of the complications associated with obesity – the physiological and pathological role of adipocytokines]. Nippon Rinsho.

[B12] Hotta K, Funahashi T, Arita Y, Takahashi M, Matsuda M, Okamoto Y, Iwahashi H, Kuriyama H, Ouchi N, Maeda K, Nishida M, Kihara S, Sakai N, Nakajima T, Hasegawa K, Muraguchi M, Ohmoto Y, Nakamura T, Yamashita S, Hanafusa T, Matsuzawa Y (2000). Plasma concentrations of a novel, adipose-specific protein, adiponectin, in type 2 diabetic patients. Arterioscler Thromb Vasc Biol.

[B13] Arita Y, Kihara S, Ouchi N, Takahashi M, Maeda K, Miyagawa J, Hotta K, Shimomura I, Nakamura T, Miyaoka K, Kuriyama H, Nishida M, Yamashita S, Okubo K, Matsubara K, Muraguchi M, Ohmoto Y, Funahashi T, Matsuzawa Y (1999). Paradoxical decrease of an adipose-specific protein, adiponectin, in obesity. Biochemical & Biophysical Research Communications.

[B14] Hotta K, Funahashi T, Bodkin NL, Ortmeyer HK, Arita Y, Hansen BC, Matsuzawa Y (2001). Circulating concentrations of the adipocyte protein adiponectin are decreased in parallel with reduced insulin sensitivity during the progression to type 2 diabetes in rhesus monkeys. Diabetes.

[B15] Maeda N, Shimomura I, Kishida K, Nishizawa H, Matsuda M, Nagaretani H, Furuyama N, Kondo H, Takahashi M, Arita Y, Komuro R, Ouchi N, Kihara S, Tochino Y, Okutomi K, Horie M, Takeda S, Aoyama T, Funahashi T, Matsuzawa Y (2002). Diet-induced insulin resistance in mice lacking adiponectin/ACRP30. Nature Medicine.

[B16] Combs TP, Berg AH, Obici S, Scherer PE, Rossetti L (2001). Endogenous glucose production is inhibited by the adipose-derived protein Acrp30. J Clin Invest.

[B17] Combs TP, Wagner JA, Berger J, Doebber T, Wang WJ, Zhang BB, Tanen M, Berg AH, O'Rahilly S, Savage DB, Chatterjee K, Weiss S, Larson PJ, Gottesdiener KM, Gertz BJ, Charron MJ, Scherer PE, Moller DE (2002). Induction of adipocyte complement-related protein of 30 kilodaltons by PPARgamma agonists: a potential mechanism of insulin sensitization. Endocrinology.

[B18] Yang WS, Jeng CY, Wu TJ, Tanaka S, Funahashi T, Matsuzawa Y, Wang JP, Chen CL, Tai TY, Chuang LM (2002). Synthetic peroxisome proliferator-activated receptor-gamma agonist, rosiglitazone, increases plasma levels of adiponectin in type 2 diabetic patients. Diabetes Care.

[B19] Naderali EK, Estadella D, Rocha M, Pickavance LC, Fatani S, Denis RG, Williams G (2003). A fat-enriched, glucose-enriched diet markedly attenuates adiponectin mRNA levels in rat epididymal adipose tissue. Clinical Science.

[B20] Adams M, Montague CT, Prins JB, Holder JC, Smith SA, Sanders L, Digby JE, Sewter CP, Lazar MA, Chatterjee VK, O'Rahilly S (1997). Activators of peroxisome proliferator-activated receptor gamma have depot-specific effects on human preadipocyte differentiation. J Clin Invest.

[B21] Kast-Woelbern HR, Dana SL, Cesario RM, Sun L, de Grandpre LY, Brooks ME, Osburn DL, Reifel-Miller A, Klausing K, Leibowitz MD (2004). Rosiglitazone induction of Insig-1 in white adipose tissue reveals a novel interplay ofperoxisome proliferator-activated receptor gamma and sterol regulatory element-binding protein in the regulation of adipogenesis. J Bio Chem.

[B22] Tschritter O, Fritsche A, Thamer C, Haap M, Shirkavand F, Rahe S, Staiger H, Maerker E, Haring H, Stumvoll M (2003). Plasma adiponectin concentrations predict insulin sensitivity of both glucose and lipid metabolism. Diabetes.

[B23] Chu NF, Spiegelman D, Yu J, Rifai N, Hotamisligil GS, Rimm EB (2001). Plasma leptin concentrations and four-year weight gain among US men. Int J Obesity.

[B24] Sinha D, Addya S, Murer E, Boden G (1999). 15-Deoxy-delta(12,14) prostaglandin J2: a putative endogenous promoter of adipogenesis suppresses the ob gene. Metabolism.

[B25] Goetze S, Bungenstock A, Czupalla C, Eilers F, Stawowy P, Kintscher U, Spencer-Hansch C, Graf K, Nurnberg B, Law RE, Fleck E, Grafe M (2002). Leptin induces endothelial cell migration through Akt, which is inhibited by PPARgamma-ligands. Hypertension.

[B26] Willson TM, Lambert MH, Kliewer SA (2001). Peroxisome proliferation-activated receptor gamma and metabolic disease. Annu Rev Biochem.

[B27] Fiedorek FT, Wilson GG, Frith L, Patel J, Abou-Donia M, Study Group-PPA20005 (2000). Monotherapy with GI262570. a tyrosine-based non-thiazolidinedione PPARγ agonist, improves metabolic control in type 2 diabetes mellitus patients [abstract]. Diabetes.

[B28] Brown KK, Faison WL, Hashim M, Harrington W, Binz J, Oliver W (2000). Antidiabetic efficacy of GI262570 in rodent models of type 2 diabetes [abstract]. Diabetes.

[B29] Suh N, Wang Y, Williams CR, Risingsong R, Gilmer T, Willson TM, Sporn MB (1999). A new ligand for the peroxisome proliferator-activated receptor-gamma (PPAR-gamma), GW347845, inhibits rat mammary carcinogenesis. Cancer Research.

[B30] Li AC, Brown KK, Silvestre MJ, Willson TM, Palinski W, Glass CK (2000). Peroxisome proliferator-activated receptor gamma ligands inhibit development of atherosclerosis in LDL receptor-deficient mice. J Clin Invest.

[B31] Chirgwin JM, Przybyla AE, MacDonald RJ, Rutter WJ (1979). Isolation of biologically active ribonucleic acid from sources enriched in ribonuclease. Biochemistry.

[B32] Bustin S (2000). Absolute quantification of mRNA using real-time reverse transcription polymerase chain reaction assays. J Mol Endocrinol.

